# The Effects of Dry-Needling Therapy on the Quality of Life in Athletes with Myofascial Pain Syndrome: Repeated Measures Design Study

**DOI:** 10.3390/jcm13174969

**Published:** 2024-08-23

**Authors:** Bojan Pavlović, Lazar Toskić, Vanja Cicović, Borislav Cicović, Veroljub Stanković

**Affiliations:** 1 Faculty of Physical Education and Sport, University of East Sarajevo, 71123 East Sarajevo, Bosnia and Herzegovina; bojan.pavlovic@ffvis.ues.rs.ba (B.P.); vanja.cicovic@ffvis.ues.rs.ba (V.C.); borislav.cicovic@ffvis.ues.rs.ba (B.C.); 2 Faculty of Sport and Physical Education, University of Priština—Kosovska Mitrovica, 38218 Leposavić, Serbia; veroljub.stankovic@pr.ac.rs; 3 Faculty of Sport, University “Union-Nikola Tesla”, 11000 Belgrade, Serbia

**Keywords:** dry needling, myofascial pain syndrome, athletes, quality of life

## Abstract

**Background:** This study aims to investigate the effects of dry-needling treatment on the quality of life in athletes with myofascial pain syndrome (MPS). **Methods:** The participants included in the study were 50 athletes (38 males and 12 females) diagnosed with MPS. The treatments were carried out in four sessions, 5/7 days apart on 55 muscles in total. A 36-item health survey (SF-36) was implemented to determine the participants’ quality of life. The chi-square test was used to determine the differences between measurements. **Results:** Dry-needling treatment has a positive influence on self-perspective of physical functioning (*p* = 0.011, on average), physical problems (*p* = 0.001, on average), emotional problems (*p* = 0.004, on average), social functioning (*p* = 0.001, on average), pain (*p* = 0.001, on average), and mental health and vitality (*p* = 0.001, on average) in athletes with MPS. The only quality-of-life dimension not influenced by the dry-needling treatment is the general health perception (*p* = 0.340, on average). **Conclusions:** Dry-needling therapy has positive effects on the perception of quality of life in athletes with MPS.

## 1. Introduction

In recent decades, clinical and scientific interest in dry-needling (DN) therapy has grown exponentially. Dry needling is a treatment modality that is minimally invasive, inexpensive, and easy to learn with proper training, while posing a low risk of procedure-related complications [1,2,3]. Dry needling is defined as a medical intervention that uses a fine filiform needle to penetrate the skin and stimulate myofascial trigger points (MTrPs), muscles, and connective tissue to treat musculoskeletal pain disorders [3,4,5]. Its effectiveness has been confirmed by numerous studies [6,7,8].

Myofascial pain syndrome (MPS) is a condition that needs to be dealt with by medical professionals worldwide. This syndrome is characterized by MTrPs and fascial constrictions, results from acute and chronic musculoskeletal pain, and often has a referred neuropathic component [9,10]. It affects more than three quarters of the world population and is one of the most important yet neglected causes of disability [11,12]. Approximately 30–93% of patients with musculoskeletal symptoms suffer from MPS, the incidence of which is higher in women [11,13]. The trapezius, rhomboid, infraspinatus, levator scapulae, and paravertebral muscles are most often affected [12].

Frequent physical activity of high volume and intensity, which is a requisite in professional sports, can imply a coinciding increase in associated risk factors, both external and internal, for the occurrence of MPS [14]. There have been numerous studies undertaken to discover the prevalence of MPS and MTrPs in athletes, as well as their diagnoses and adequate therapy [15,16,17]. Trigger points are considered as generators of peripheral pain in the conditions of general or regional musculoskeletal pain, and their biochemical milieu has revealed the release of inflammatory mediators in combination with low pH and local ischemia [18,19]. A high MTrPs prevalence varying from 13% to 30% has been recorded in gastrocnemius muscle in athletes [16]. In addition, musculoskeletal changes in the lower limbs can account for up to one third of medical consultations in sports [20]. Indeed, gastrocnemius injuries are considered the most common conditions found in the lower limbs in athletes, with a 39% to 46% prevalence occurring in training or competition [16]. Anterior knee pain (AKP), a widespread problem among young athletes, also has myofascial trigger points involved among its other etiologies. In the research by Rozenfeld et al. [21], it was shown that subjects with AKP had a higher prevalence of MTrPs in hip and thigh muscles, which indicates an association between MTrP and AKP. Also, athletes from sports involving a wide variety of throwing actions, in which the shoulder is under high demand due to repeated overhead movements at high speed, are susceptible to MPS. Such repetitive movements cause symptoms of overuse injuries and myofascial pain syndrome. Recent studies have reported the prevalence of acute or chronic shoulder pain in handball players to be between 36% and 44.2%. Athletes with glenohumeral internal rotation deficit and increased external rotation are at greater risk of suffering from shoulder pain. Also, it was shown that there is a connection between pain and changes in glenohumeral rotation range of motion in handball players, and the presence of myofascial trigger points (MTrPs) in the teres major muscle [22].

Previous studies have investigated the influence of dry-needling treatment on myofascial pain syndrome, and the results showed that dry-needling treatment is effective in relieving pain and improving the quality of life of patients with MPS [8,23,24]. Also, so far, there have been studies that investigated the influence of dry-needling treatment on various properties in athletes with myofascial pain syndrome [22,25,26]; however, none of these studies investigate the influence of dry-needling treatment on quality of life in athletes with MPS. Accordingly, this study aims to investigate the effects of dry-needling treatment on the quality of life in athletes with MPS. It is hypothesized that dry-needling therapy will have a significant impact on the quality of life in athletes with MPS.

## 2. Materials and Methods

### 2.1. Subjects

In this study, the convenience sampling method was used. The participants included in the study were 50 athletes: 38 males (age: 23 ± 2.4 years, body height = 181.6 ± 4.2 cm, body weight = 80.5 ± 4.1 kg, on average) and 12 females (age: 22.7 ± 1.9 years, body height = 170.4 ± 4.1 cm, body weight = 68.6 ± 5.2 kg, on average). All participants were athletes with a minimum of 5 years of experience in national and international competitions in the following sports: football (8), basketball (4), volleyball (4), handball (8), combat sports (7), swimming (5), tennis (7), and track and field (7). All participants had been diagnosed with MPS, which was one of the criteria for participating in the study. MPS diagnostic criteria were [23]:

- localized spontaneous pain;
- spontaneous pain or altered sensations in the expected referred pain area for a given trigger point;
- taut, palpable band in accessible muscle;
- exquisite, localized tenderness in precise point along taut band;
- some measurable degree of reduced range of movement;
- reproduction of spontaneously perceived pain and altered sensations by pressure on trigger point;
- elicitation of a local twitch response of muscular fibers by transverse “snapping” palpation or by needle insertion into the trigger point;
- pain relief obtained by muscle stretching or injection of trigger point.

Besides actively participating in sports training and competitions and MPS diagnosis, the additional criteria for participating in the study was the absence of other illnesses or injuries. All participants were adults who, having been familiarized with the study’s aim and procedures, voluntarily agreed to participate in the study providing signed written consent. All measurements were made in accordance with the Declaration of Helsinki and were approved by the Ethical Committee of the Faculty of Physical Education and Sport (1489/24).

### 2.2. Procedures

The research was conducted using a repeated-measures design in which participants were required to visit the research laboratory on four different days/sessions, separated by a rest period of 5–7 days [27]. The study was conducted from the 15 January–30 February 2024. Before the dry-needling treatment, each participant was informed about the procedure, its possible side effects (slight pain and bleeding, bruising) and the method of recovery (warm shower or gentle heating of the treated area), and was instructed to relax and stay hydrated. The participant was placed comfortably in the proper position for the body part to be treated, a lying or sitting position, depending on the treated muscle. The part to be treated was freed from clothing. After a palpatory examination and localization of the trigger point (tender spots) [28,29], the therapist positioned the needle and the guide tube with the hand used for needling. The penetration site having been disinfected, the sterile acupuncture needle was inserted into the epidermal skin layer with a quick stroke, and the guide tube was discarded [3].

The dominant hand was used to insert the needle perpendicular to the muscle, either superficially into the subcutaneous tissue or deep into the muscle, depending on the therapist’s diagnosis, to penetrate the trigger point [3]. The needle was left in place for 10 to 20 min, during which the therapist slightly changed the position of the needle several times to further stimulate the tissue for better effectiveness [4]. The needle was then removed and disposed of in an appropriate sharp waste container. The injection site was cleaned with a sterile swab. All treatments and diagnoses were conducted by a licensed dry-needling therapist with a minimum of 10 years of experience. The following muscles were treated (the number in brackets presents the treated muscles in total): m. Latissimus dorsi (6), m. Infraspinatus (9), m. Trapezius (9), m. Deltoideus (7), m. Tensor fasciae latae (6), m. Adductor longus, magnus et brevis (8), m. Iliopsoas (2), m. Rectus femoris (3), m. Biceps femoris (2), m. Vastus lateralis et medialis (1), m. Semimembranosus (1), m. Semitendinosus (1), m. Coracobrachialis (1), m. Tibialis posterior (1), m. Sartorius (4), m. Quadratus lumborum (3), m. Iliocostalis (3), m. Pectineus (1), m. Gracilis (7), m. Tibialis anterior (1), m. Gastrocnemius et Soleus (5), m. Peroneus longus et brevis (4), m. Pectoralis major (4), m. Pectoralis minor (1), m. Teres minor (1), m. Teres major (2), m. Triceps brachii (2), m. Pectineus (1), m. Biceps brachii (3), m. Brachialis (1), m. Brachioradialis (3), m. Supinator (4), m. Flexor carpi radialis (1), m. Pronator teres (4), m. Tibialis anterior (3), m. Multifidi (1), m. Triceps surae (2), m. Erector spinae (2), m. Levator scapulae (5), m. Subscapularis (3), m. Multifidus (2), m. Gluteus maximus, medius et minimus (2), m. Supraspinatus (2), m. Rhomboideus (3), m. Sternocleidomastoideus (1), m. Extensor carpi ulnaris (1), m. Pronator quadratus (1), and m. Piriformis (1).

To determine the participants’ quality of life, a 36-item health survey (SF-36) was implemented [30,31]. The SF-36 questionnaire contains multi-item scales measuring eight generic health concepts (Table 1). Patients were evaluated two times, before the first treatment and immediately after the last treatment [23].

### 2.3. Statistical Analysis

Adequate methods of classic descriptive statistics were used to describe the sample, chosen in accordance with the type of data: absolute and relative (frequency, %) values. The chi-square test (*X*^2^-test) was implemented to determine the differences between the measurements. The level of statistical significance was 95% with *p* < 0.05 [32]. All the statistical procedures were performed in the SPSS19 (IBM, Armonk, NY, USA) program.

## 3. Results

Table 2 represents the results of self-rating of health before and after the therapy. Only one respondent rated their health as “excellent” before therapy. After therapy, 56% of the respondents rated their health as “very good”, while none of the participants rated their health as poor (*p* = 0.001).

There was a statistically significant improvement in activity after therapy (Supplementary material, Table S1). Before therapy, 56% of respondents reported very limited vigorous activities, while after therapy only 4% rated their activities as limited (*p* = 0.001). Twenty participants (40%) of respondents reported very limited moderate activities before therapy, compared to 12% after therapy (*p* = 0.001), 40% of respondents had severe limitations in lifting or carrying groceries before therapy, while there were no such reports after therapy (*p* = 0.001). A total of 22% of respondents had severe limitations in climbing a few flights of stairs before therapy, while there were no such reports after therapy (*p* = 0.001). Climbing one flight of stairs with severe limitations was experienced by 8% of respondents before therapy and none after therapy (*p* = 0.048). Before therapy, 28% of respondents had great limitations while bending, kneeling or stooping, while after therapy there were no such responses (*p* = 0.001). Walking more than 1.6 km with severe limitations was experienced by 16 respondents before therapy and two after therapy (*p* = 0.001), while 16% of respondents were severely limited in walking several blocks before therapy but there were no such respondents after therapy (*p* = 0.011). Walking one block with severe limitation was experienced by 8% of respondents before therapy and none after therapy (*p* = 0.043). A total of 14% of respondents before therapy and none after therapy had severe limitations while bathing (*p* = 0.006).

Before therapy, 78% of respondents had to reduce the time they spent at work, but after therapy, this percentage was 34% (*p* = 0.001) (Table 3). Before therapy, 70% of respondents accomplished less than they wanted, while after therapy, this percentage dropped to 32% (*p* = 0.001). Before therapy, 90% of respondents reported some type of work limitation, and after therapy, the percentage was 48% (*p* = 0.001). A total of 82% of respondents had difficulty performing work or other activities before therapy, compared to 40% after therapy (*p* = 0.001).

Prior to therapy, 58% of respondents had to cut down the amount of time spent at work due to problems related to emotional health, while after therapy, this percentage fell to 30% (*p* = 0.004) (Table 4). Before therapy, 54% achieved less than they wanted, while after therapy there were only 28% of such responses (*p* = 0.007). A total of 64% of respondents reported a drop in carefulness while working before therapy, compared to the percentage of 16% reported after therapy (*p* = 0.001).

The percentage of professional athletes whose social activities were extremely affected before therapy was 16%, while after therapy none of the respondents reported having severe problems (*p* = 0.001) (Table 5). The analysis of the interference of physical health and emotional problems with the respondents’ social activities revealed that the highest percentage (68%) had problems some of the time before therapy, compared to 46% who reported after therapy that they had such problems a little of the time, which is a statistically significant improvement (*p* = 0.001).

The highest percentage of professional athletes had moderate (52%) and severe body pain (38%) before therapy (Table 6). Following therapy, they mostly reported mild (40%) and very mild body pain (26%), and a statistically significant improvement after therapy was observed (*p* = 0.001). In the self-reports of professional athletes prior to therapy, pain most often had a considerable (40%) and moderate (46%) effect on the performance of normal work. After therapy, there was a statistically significant improvement, with pain mainly interfering with the performance of normal work slightly (66%) or moderately (28%) (*p* = 0.001).

The chi-square test showed a statistically significant improvement after therapy regarding mental health and vitality (Supplementary material, Table S2). The highest percentage of respondents (38%) felt before therapy that they were full of energy some of the time, while after therapy, 58% responded that they felt full of energy most of the time (*p* = 0.002). A total of 46% of respondents felt very nervous some of the time before therapy, while after therapy the highest percentage of respondents (72%) felt very nervous a little of the time (*p* = 0.001). Before therapy, 28% and 26% felt miserable most of the time and a little of the time, respectively, while after therapy, 58% of respondents were in such a mood for short durations only (*p* = 0.001). Also, the feeling of peace and calm was felt by the highest percentage of respondents a little of the time before therapy (48%), while after therapy, 58% had this feeling most of the time. Before therapy, the highest percentage of respondents had a lot of energy for short durations (58%), while after therapy, the same percentage felt very energetic most of the time. A total of 52% of respondents felt downhearted and blue some of the time before therapy, and after therapy 70% felt like this for a little of the time (*p* = 0.001). A total of 52% of respondents felt worn out some of the time before therapy, while this feeling was reported as felt a little of the time by 62% of respondents after therapy (*p* = 0.001). Before therapy, 46% of professional athletes self-rated themselves as happy a little of the time, but after therapy, 54% were happy most of the time (*p* = 0.001). Before therapy, 46% of respondents felt tired some of the time, and after the therapy, 72% felt tired a little of the time (*p* = 0.001).

No statistically significant difference was found in the before- and after-therapy statements on whether the respondents got sick a little easier than others (*p* = 0.898) (Table 7). The statements on being as healthy as other people they knew did not statistically change after therapy compared to before therapy (*p* = 0.305). The respondents’ expectations that their health would deteriorate also did not change, as their statements before and after therapy were not statistically significantly different (*p* = 0.157). However, a statistically significant difference was found in the statements about health before and after therapy (*p* = 0.001). After therapy, most respondents (42) generally considered their health to be excellent compared to the same responses given before therapy (28).

## 4. Discussion

This study aimed to investigate the effects of dry-needling treatment on the quality of life in athletes with MPS. To the best of the authors’ knowledge, this is the first study that dealt with this problem. The results of this study could lead to important information about the influence of dry-needling treatment on functional status, wellbeing, and general health perception in athletes with MPS.

The results of the present study revealed that dry-needling treatment significantly positively influences the quality of life in athletes with MPS. Namely, dry-needling treatment has been shown to have a positive influence on self-perspective of physical functioning (*p* = 0.011, on average), physical problems (*p* = 0.001, on average), emotional problems (*p* = 0.004, on average), social functioning (*p* = 0.001, on average), pain (*p* = 0.001, on average), and mental health and vitality (*p* = 0.001, on average) in athletes with MPS. The only quality-of-life dimension which is not influenced by the dry-needling treatment is the general health perception, where three out of four statements were not significantly changed after the treatments (*p* = 0.340, on average).

Based on the research results presented above and the correlations with the research results of other authors in this field [22,25,26], it can be concluded that the working hypotheses were confirmed, suggesting that dry-needling therapy had a significant positive effect on relieving myofascial pain syndrome in athletes, as well as that it had a positive effect on the quality of life and daily MPS.

Myofascial pain syndrome (MPS) is described as a symptom complex in the muscular, sensory, motor, and autonomic nervous systems caused by stimulation of myofascial trigger points (MTrPs). This condition is believed to be caused by muscle overuse, trauma, or psychological stress [15], which is a frequent occurrence in sports. A variety of traumas, such as contusions, sprains, and strains, can cause acute MTrPs. These trigger points can be activated by repetitive micro-injuries, including muscle overload and overuse, which often lead to chronic MTrPs. According to the principle of muscle fiber size, a small load force acting on a joint or the spine for a long time can cause myalgia. When maintaining a sustained static load, the smaller Type I muscle fibers are first to recruit and last to relax. Therefore, this type, sometimes called Cinderella fibers, can be easily injured. The “Cinderella” hypothesis of an energy crisis advocated for by Simmons and Travell [33] claims that increased energy consumption, relaxation disorders, reduced energy recovery, and impaired blood flow lead to the contraction of muscle fibers and the creation of trigger points. As a result, chronic myofascial pain occurs.

However, what causes an otherwise normal muscle to experience an energy crisis? In postural muscles (such as deep neck muscles), motor units are recruited by rotation rather than size, allowing them to contract and then relax in a displacement-like manner. Because rotational recruitment excludes the presence of Cinderella units (muscle type I fibers), the Cinderella hypothesis may not be a good model for myofascial pain in postural muscles, despite observations that these muscles are involved in myofascial pain more often than others. In the above-mentioned “Cinderella” hypothesis, increased energy demand and reduced oxygen supply during contraction lead to the creation of trigger points. However, the group of authors hypothesized that the relaxation time of the motor units could be as important as the contraction time since it is during relaxation that the motor units benefit from improved microvascular blood flow and are therefore able to replenish their oxygen and glucose supply and get rid of harmful metabolites. Minerbi and Vulfsons presented a shift model [34]. The proposed model implies that the energy crisis could be a threshold phenomenon when the relaxation/contraction duration ratio falls below a certain value. They hypothesized that motor unit relaxation time may be important in developing an energy crisis. They observed that increased muscle load and muscle weakening lead to shorter relative relaxation times for each motor unit and, consequently, to an energy crisis, thus potentially resulting in the development of myofascial pain.

MTrPs often occur in this type of muscle fiber with multiple ratios within the muscle to maintain postural position. In this case, the upper trapezius and levator scapulae can also be easily injured, i.e., MTrPs can often be formed in these two muscles [2]. Age-related degeneration of the musculoskeletal system, with gradual loss of myofascial flexibility, causes susceptibility and eventually results in active MTrPs. Certain compressions or non-bacterial inflammations of the spinal column can irritate the nerve root, causing sensitization of the spinal segment so that MTrPs get activated in the innervated muscles. Emotional stress can increase sympathetic nervous system activity and cause sleep deprivation, significantly intensifying muscle tension and fatigue, introducing anxiety and insomnia, and lowering the pain threshold. Endocrine and metabolic deficiencies and thyroid and estrogen insufficiency can irritate latent MTrPs into action. Meanwhile, nutritional vitamin and mineral deficiencies can prolong the activity of MTrPs [35].

As this (and some previous) study showed, dry-needling treatment has a positive effect on various properties in patients with MPS. However, the mechanism is still not completely clear. Simons proposed the integrated trigger point hypothesis, according to which an excessive amount of acetylcholine is thought to be released at the motor endplate. The acetylcholinesterase inhibition at the endplate leads to increased motor plate activity, in turn causing continuous release of calcium ions (Ca^+2^), which may explain the presence of a trigger point. It is also possible that trigger points develop due to repetitive low stress or moderate overuse, which is the reasoning behind the Cinderella hypothesis previously explained. Henneman’s size principle states that smaller, Type I muscle fibers are recruited first and relaxed last. These “Cinderella” fibers are constantly recruited so that the large motor fibers do not have to work as hard. Hypoxia can develop due to long-term muscle contractions, leading to a drop in the pH value, which then can trigger the release of inflammatory mediators and neurotransmitters such as calcitonin gene-related peptide, prostaglandins, substance P, 5-HT, and ATP among others. This can cause increased nociceptive spinal input, which can lead to peripheral and central sensitization. Sometimes the application of deep dry needling to a trigger point elicits a local twitch response (LTR), the occurrence of which is yet not well understood. After LTR, a decrease was observed in the concentrations of calcitonin gene-related peptide, substance P, interleukins, and cytokines [36,37,38].

The results of the present study can be explained by previous statements. Namely, dry-needling treatment positively impacts the functional problems caused by MPS through physiological factors. On the other side, it has been shown that dry-needling therapy has a significant influence on pain reduction [8], which leads to improved mental health [39]. Mental health generally has an impact on quality of life [40], and it can also play a key role in myofascial pain in athletes and sports performance in general [41]. In conclusion, dry-needling therapy has positive effects on functional status and wellbeing in athletes with MPS through physiological and psychological factors. The only self-perceived dimension that is not influenced by dry needling is general health perception. The reason for this phenomenon may be that athletes who regularly participate in sports activities for a long period, besides MPS, have a high level of their general health and perception of general health at the baseline.

The results of this study indicate that dry-needling therapy is effective in reducing discomfort regarding MPS and improving the general quality of life in athletes with MPS. Accordingly, dry-needling treatment can be recommended as a regular therapy, alongside other types of therapies, in treatment of MPS in athletes.

The main limitation of the study is the dry-needling therapy dosage. There is currently no consensus on the most appropriate technique and dosage of dry needling. The authors decided to conduct the study with a relatively small (optimal) number of treatments, depending on subjects’ availability for participating in the study. Another study limitation is the non-use of ultrasonography, as a method for determining therapeutic accuracy. The ultrasonography was not used due to limited access to this device. Also, the authors did not consider the influence of verbal suggestions to patients on post-needling soreness and pain, which could influence the general perception of quality of life. Finally, the implemented questionnaire is one of the study’s limitations, since some items are not completely appropriate for the athletes. Further studies should focus on implementing different treatment procedures (number of therapies, rest between therapies, verbal suggestions etc.), and usage of additional instruments to collect more important information about the influence of dry-needling treatment on the quality of life in athletes with MPS.

## 5. Conclusions

The results of the present study showed that dry-needling therapy significantly influences the quality of life in athletes with MPS. Namely, dry-needling treatment has been shown to have a positive influence on self-perspective of functional status (physical functioning, physical problems, emotional problems, social functioning, pain), and wellbeing (mental health and vitality) in athletes with MPS. The only quality-of-life dimension that is not influenced by the dry-needling treatment is general health perception.

## Figures and Tables

**Table 1:** Dimensions of the SF36 questionnaire.

Area	Dimensions	Number of Questions
Functional status	Physical functioning	10
	Social functioning	2
	Role limitations (physical problems)	4
	Role limitations (emotional problems)	3
Well being	Mental health	5
	Vitality	4
	Pain	2
Overall evaluation of the health	General health perception	5
	Health change *	1
Total		36

Legend: SF36—36 item health survey questionnaire; Health change * is not included in eight dimensions nor it is scored.

**Table 2:** Self-rating of health before and after therapy.

	Before Therapy	After Therapy	*X* ^2^	*p*
Excellent	1 (2%)	2 (4%)	18.171	0.001
Very good	13 (26%)	28 (56%)		
Good	21 (42%)	20 (40%)		
Poor	15 (30%)	0 (0%)		

**Table 3:** Role limitations (physical problems)—During the past 4 weeks, have you had any of the following problems with your work or other regular daily activities as a result of your physical health?

		Before Therapy	After Therapy	*X* ^2^	*p*
Shortened time spent working	NO	11 (22%)	33 (66%)	19.6	0.001
	YES	39 (78%)	17 (34%)		
Accomplished less than wanted	NO	15 (30%)	34 (68%)	14.5	0.001
	YES	35 (70%)	16 (32%)		
Limited in the kind of work or other activities	NO	5 (10%)	26 (52%)	20.6	0.001
	YES	45 (90%)	24 (48%)		
Difficulty performing the work	NO	9 (18%)	30 (60%)	18.8	0.001
	YES	41 (82%)	20 (40%)		

**Table 4:** Role limitations (emotional problems)—During the past 4 weeks, have you had any of the following problems with your work or other regular daily activities as a result of your emotional problems?

		Before Therapy	After Therapy	*X* ^2^	*p*
Shortened time spent working	NO	21 (42%)	35 (70%)	7.96	0.004
	YES	29 (58%)	15 (30%)		
Accomplished less than wanted	NO	23 (46%)	36 (72%)	7.06	0.007
	YES	27 (54%)	14 (28%)		
Lack of carefulness performing work	NO	18 (36%)	36 (72%)	24	0.001
	YES	32 (64%)	14 (28%)		

**Table 5:** Social functioning.

		Before Therapy	After Therapy	*X* ^2^	*p*
During the past 4 weeks, to what extent have your physical health or emotional problems interfered with your normal activities with friends, neighbors, or groups?	Not at all	11 (22.0%)	13 (26.0%)	11.735	0.001
	Slightly	9 (18.0%)	21 (42.0%)		
	Moderately	22 (44.0%)	16 (32.0%)		
	Extremely	8 (16.0%)	0 (0.0%)		
During the past 4 weeks, how much of the time have your physical health or emotional problems interfered with your social activities?	Most of the time	12 (24%)	3 (6%)		
	A little of the time	3 (6%)	32 (46%)	52.653	0.001
	Some of the time	34 (68%)	7 (14%)		
	None of the time	1 (2%)	8 (16%)		

**Table 6:** Pain.

		Before Therapy	After Therapy	*X* ^2^	*p*
How much bodily pain have you had during the past 4 weeks?	None	0 (0%)	3 (6%)	42.770	0.001
	Very mild	0 (0%)	13 (26%)		
	Mild	3 (6%)	20 (40%)		
	Moderate	26 (52%)	12 (24%)		
	Severe	19 (38%)	2 (4%)		
	Very severe	2 (4%)	0 (0%)		
During the past 4 weeks, how much did the pain interfere with your normal work?	A little bit	8 (16%)	33 (66%)	32.433	0.001
	Moderately	23 (46%)	14 (28%)		
	Quite a bit	20 (40%)	3 (6%)		
	Extremely	2 (4%)	0 (0%)		

**Table 7:** General health perception.

		Before Therapy	After Therapy	*X* ^2^	*p*
I seem to get sick a little easier than other people	Mostly true	13 (26%)	13 (26%)	0.559	0.898
	Don’t know	22 (44%)	20 (40%)		
	Mostly false	12 (24%)	12 (24%)		
	Definitely false	3 (6%)	5 (10%)		
I am as healthy as anybody I know	Definitely true	1 (2%)	1 (2%)	3.621	0.305
	Mostly true	14 (28%)	22 (44%)		
	Don’t know	18 (36%)	17 (34%)		
	Mostly false	17 (34%)	10 (20%)		
I expect my health to get worse	Mostly true	2 (4%)	4 (8%)	5.209	0.157
	Don’t know	19 (38%)	9 (18%)		
	Mostly false	25 (50%)	32 (64%)		
	Definitely false	4 (8%)	5 (10%)		
My health is excellent	Definitely true	0 (0%)	2 (4%)	21.891	0.001
	Mostly true	28 (56%)	42 (84%)		
	Don’t know	5 (10%)	6 (12%)		
	Mostly false	17 (34%)	0 (0%)		

## Data Availability

The raw data supporting the conclusions of this article will be made available by the authors on request.

## Supplementary Materials

The following supporting information can be downloaded at: https://www.mdpi.com/article/10.3390/jcm13174969/s1, Table S1: Physical functioning—The following items are about activities you might do during a typical day. Does your health now limit these activities? If so, how much?; Table S2: Mental health and vitality.
