# Supplementary material for: The Effects of Dry-Needling Therapy on the Quality of Life in Athletes with Myofascial Pain Syndrome: Repeated Measures Design Study
Source: J Clin Med. 2024 Aug 23;13(17):4969. doi: 10.3390/jcm13174969 (PMC11395707; doi:10.3390/jcm13174969)
Supplement: Supplementary file 1 [file jcm-13-04969-s001.zip › jcm-3138642-supplementary.pdf]

Table S1. Physical functioning – The following items are about activities you might do during a typical day. Does your health now limit these activities? If so, how much?

		Before therapy	After therapy	X^2	p
**Vigorous activities**	**Yes, limited a lot**	28 (56%)	2 (4%)	**37.347**	**0.001**
	**Yes, limited a little**	22 (44%)	37 (74%)		
	**No, not limited at all**	0 (0%)	11 (22%)		
**Moderate activities**	**Yes, limited a lot**	20 (40%)	6 (12%)	**13.819**	**0.001**
	**Yes, limited a little**	23 (58%)	24 (48%)		
	**No, not limited at all**	7 (14%)	20 (40%)		
**Lifting or carrying groceries**	**Yes, limited a lot**	20 (40%)	0 (0%)	**25.154**	**0.001**
	**Yes, limited a little**	17 (34%)	26 (52%)		
	**No, not limited at all**	13 (26%)	24 (48%)		
**Climbing several flights of stairs**	**Yes, limited a lot**	11 (22%)	0 (0%)	**17.453**	**0.001**
	**Yes, limited a little**	21 (42%)	15 (30%)		
	**No, not limited at all**	18 (36%)	35 (70%)		
**Climbing one flight of stairs**	**Yes, limited a lot**	4 (8%)	0 (0%)	**6.071**	**0.048**
	**Yes, limited a little**	17 (34%)	12 (24%)		
	**No, not limited at all**	29 (58%)	38 (76%)		
**Bending, kneeling, or stooping**	**Yes, limited a lot**	14 (28%)	0 (0%)	**16.745**	**0.001**
	**Yes, limited a little**	16 (32%)	26 (52%)		
	**No, not limited at all**	20 (40%)	24 (48%)		
**Walking more than a mile**	**Yes, limited a lot**	16 (32%)	2 (4%)	**13.3**	**0.001**
	**Yes, limited a little**	14 (28%)	19 (38%)		
	**No, not limited at all**	20 (40%)	29 (58%)		
**Walking several blocks**	**Yes, limited a lot**	8 (16%)	0 (0%)	**8.97**	**0.011**
	**Yes, limited a little**	13 (26%)	13 (26%)		
	**No, not limited at all**	29 (58%)	37 (74%)		
**Walking one block**	**Yes, limited a lot**	4 (8%)	0 (0%)	**6.27**	**0.043**
	**Yes, limited a little**	13 (26%)	8 (16%)		
	**No, not limited at all**	33 (66%)	42 (84%)		
**Bathing or dressing yourself**	**Yes, limited a lot**	7 (14%)	0 (0%)	**10.259**	**0.006**
	**Yes, limited a little**	28 (56%)	24 (48%)		
	**No, not limited at all**	15 (30%)	26 (52%)		

Table S2. Mental health and vitality

		Before therapy	After therapy	X²	P
**Did you feel full of pep?**	**All of the time**	2 (4%)	7 (14%)	**20.253**	**0.001**
	**Most of the time**	12 (24%)	29 (58%)		
	**A little of the time**	16 (32%)	8 (16%)		
	**Some of the time**	19 (38%)	6 (12%)		
	**None of the time**	1 (2%)	0 (0%)		
**Have you been very nervous person?**	**All of the time**	1 (2%)	0 (0%)	**26.948**	**0.001**
	**Most of the time**	12 (24%)	2 (4%)		
	**A little of the time**	14 (28%)	36 (72%)		
	**Some of the time**	23 (46%)	9 (18%)		
	**None of the time**	0 (0%)	3 (6%)		
**Have you felt so down in the dumps that nothing could cheer you up?**	**All of the time**	1 (2%)	0 (0%)	**21.585**	**0.001**
	**Most of the time**	14 (28%)	2 (4%)		
	**A little of the time**	13 (26%)	29 (58%)		
	**Some of the time**	17 (34%)	8 (16%)		
	**None of the time**	5 (10%)	11 (22%)		
**Have you felt calm and peaceful?**	**All of the time**	1 (2%)	5 (10%)	**27.77**	**0.001**
	**Most of the time**	8 (16%)	29 (58%)		
	**A little of the time**	24 (48%)	6 (12%)		
	**Some of the time**	16 (32%)	10 (20%)		
	**None of the time**	1 (2%)	0 (0%)		
**Did you have a lot of energy?**	**All of the time**	0 (0%)	5 (10%)	**29.418**	**0.001**
	**Most of the time**	10 (20%)	29 (58%)		
	**A little of the time**	29 (58%)	6 (12%)		
	**Some of the time**	11 (22%)	10 (20%)		
**Have you felt downhearted and blue?**	**All of the time**	1 (2%)	0 (0%)	**39.75**	**0.001**
	**Most of the time**	13 (26%)	1 (2%)		
	**A little of the time**	9 (18%)	35 (70%)		
	**Some of the time**	26 (52%)	8 (16%)		
	**None of the time**	1 (2%)	6 (12%)		
**Have you felt worn out?**	**All of the time**	7 (14%)	0 (0%)	**35.185**	**0.001**
	**Most of the time**	14 (28%)	4 (8%)		
	**A little of the time**	9 (18%)	31 (62%)		
	**Some of the time**	26 (52%)	8 (16%)		
	**None of the time**	0 (0%)	7 (14%)		
**Have you been a happy person?**	**All of the time**	2 (4%)	5 (10%)	**16.722**	**0.001**
	**Most of the time**	10 (20%)	27 (54%)		
	**A little of the time**	23 (46%)	9 (18%)		
	**Some of the time**	15 (30%)	9 (18%)		
**Have you felt tired?**	**All of the time**	16 (32%)	0 (0%)	**37.617**	**0.001**
	**A little of the time**	10 (20%)	36 (72%)		
	**Some of the time**	23 (46%)	10 (20%)		
	**None of the time**	1 (2%)	4 (8%)		
